# Septic Vasculitis as a Manifestation of Invasive Infection by Community-Acquired Methicillin-Resistant Staphylococcus aureus: A Pediatric Case Report

**DOI:** 10.7759/cureus.91135

**Published:** 2025-08-27

**Authors:** Ana Campos

**Affiliations:** 1 Pediatrics, Universidad de Valparaíso, Valparaíso, CHL; 2 Pediatrics, Hospital Gustavo Fricke, Viña del Mar, CHL

**Keywords:** bacteremia, child, community-acquired infection, invasive bacterial infection, methicillin-resistant staphylococcus aureus (mrsa), pediatric, septic vasculitis

## Abstract

Septic vasculitis (SV) is an uncommon complication of bacterial sepsis, characterized by inflammation and thrombosis of small- and medium-sized blood vessels. Although it is most often associated with meningococcemia, cases linked to *Staphylococcus aureus* have been reported. Presented is the case of a previously healthy adolescent who developed SV as an initial manifestation of community-acquired methicillin-resistant *Staphylococcus aureus* (CA-MRSA) bacteremia.

The patient presented with fever, abdominal pain, and purpuric skin lesions, without any known exposure to typical risk factors. Initial clinical suspicion included loxoscelism, a necrotic arachnidism caused by *Loxosceles* spider bites, based on initial skin lesion morphology. However, further evaluation ruled out loxoscelism and autoimmune etiologies. Subsequent investigations confirmed MRSA infection through blood cultures, while skin biopsy revealed histological features of cutaneous vasculitis, establishing the diagnosis of SV. Imaging identified secondary infectious foci in the spleen, kidneys, and bone, though no surgical intervention was necessary.

The case was managed with targeted antibiotic therapy, resulting in complete clinical recovery. This case underscores the importance of recognizing SV as a rare but serious initial manifestation of invasive bacterial infection in children and highlights the diagnostic value of skin findings in febrile illnesses, emphasizing the need for prompt and comprehensive etiological investigation.

## Introduction

Cutaneous vasculitis remains a broad and varied group of conditions mainly involving the skin, characterized histopathologically by inflammation and necrosis of blood vessels. These conditions may evolve as primary idiopathic processes or occur secondary to underlying disease, medication, or infection [1]. 

Septic vasculitis (SV), a form of secondary vasculitis, occurs in the setting of bacteremia or sepsis and presents clinically as purpuric, blistering, or necrotic skin lesions [2]. A wide range of infectious agents, including viruses, bacteria, and parasites, have been implicated. Although rare, SV can be the initial manifestation of a life-threatening systemic infection, and its misdiagnosis as primary vasculitis may delay appropriate treatment. In such cases, corticosteroids, commonly used for primary vasculitis, can worsen outcomes [3].

Methicillin-resistant *Staphylococcus aureus* (MRSA), traditionally a hospital-associated pathogen, has become increasingly prevalent in the community, causing a wide spectrum of infections, from mild skin and soft tissue disease to invasive conditions such as bacteremia, endocarditis, osteomyelitis, and necrotizing pneumonia. It also triggers the activation of the immune system, which is fairly responsible for toxic shock syndrome. However, its association with vasculitis remains poorly documented [4,5].

Case reports in adults have described cutaneous vasculitis secondary to *S. aureus* bacteremia, diagnosed based on clinical and microbiological findings [6,7]. Pediatric reports are particularly scarce. Wall et al. described a preschool-aged boy with community-acquired MRSA (CA-MRSA) sepsis who developed early retiform purpura, petechiae, vesiculopustules, and hemorrhagic bullae. Skin biopsy confirmed the diagnosis of cutaneous SV. This case underscores that septic vasculopathy can be rapidly progressive and life-threatening [8].

In parts of South America, including Argentina, Peru, Chile, and Brazil, loxoscelism, caused by bites of *Loxosceles* spiders, is a significant public health concern. Its venom is dermonecrotic and viscerotoxic, leading to two clinical forms: a mild, localized cutaneous form with necrotic skin lesions that mimic vasculitic processes and a severe visceral form with systemic symptoms like hemolysis, jaundice, and renal failure [9]. 

This is a case report of a previously healthy adolescent who developed biopsy-proven SV secondary to CA-MRSA bacteremia. The clinical, microbiological, and histopathological findings are presented to contribute to the medical literature on this rare but potentially fatal presentation.

## Case presentation

**Figure 1:**
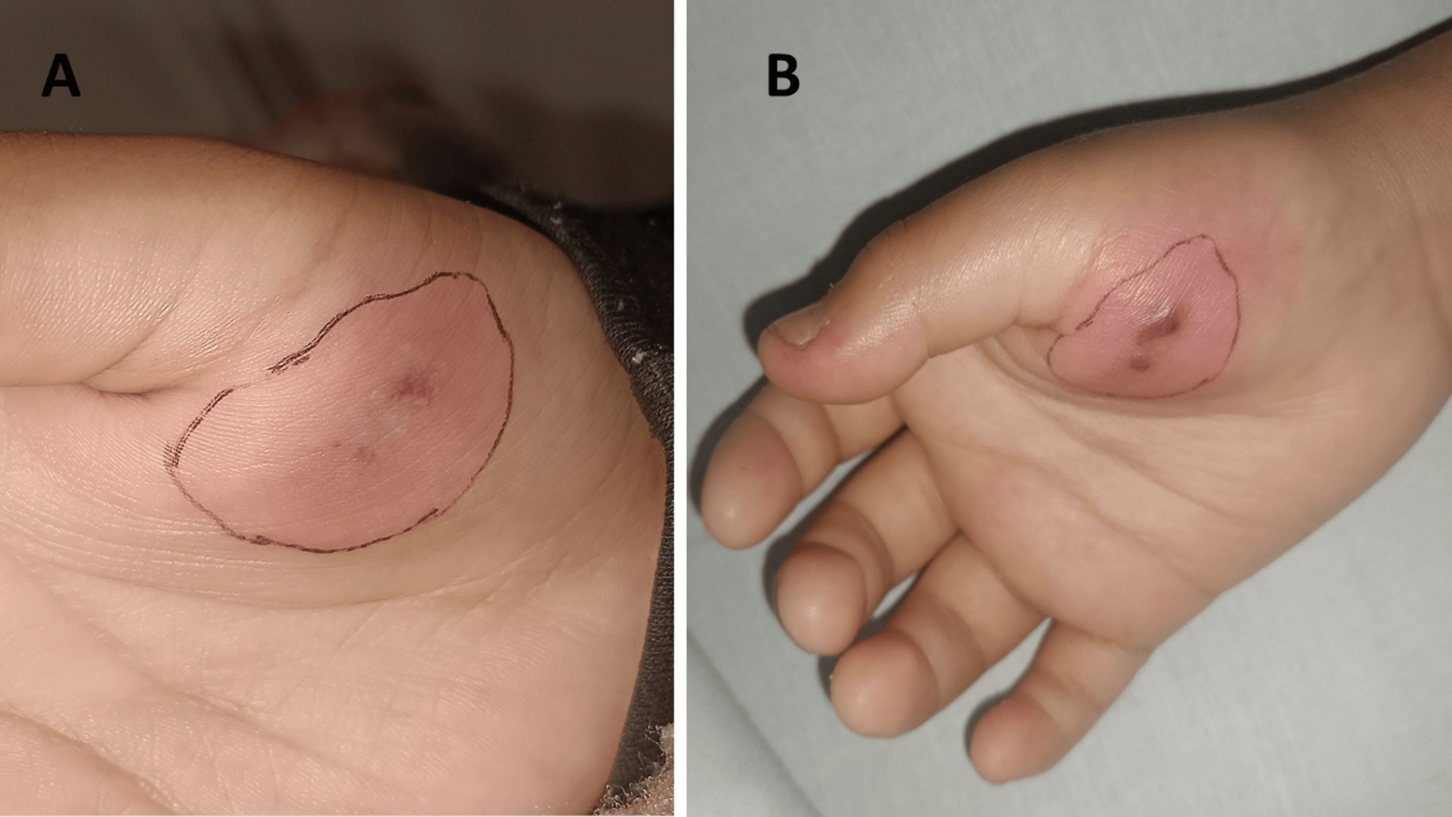
Initial cutaneous findings on the right hand at presentation: (A) erythema with a slight central violaceous coloration on the hypothenar region of the hand and (B) progression to palpable purpura in the same region

A previously healthy 12-year-old male adolescent, residing in a rural area of Quilpué, Chile, with a history of potential exposure to *Loxosceles laeta* spiders, developed localized pain and erythema in the hypothenar region of his right hand on February 16, at approximately 8:00 AM. Within hours, the skin lesion evolved to a violaceous plaque, and he developed fever up to 39°C, abdominal pain, vomiting, and diffuse lower extremities pain. These symptoms prompt consultation at the local hospital the same day at 8:00 PM, approximately 12 hours after symptom onset. He was hemodynamically stable with normal vital signs. Examination revealed a violaceous cutaneous lesion localized on his right hand (Figure 1), with no additional significant findings. Laboratory tests performed at that time showed moderate leukocytosis (15,200/uL; neutrophils 91%), mildly elevated C-reactive protein (CRP) (2.6 mg/dL), and mild proteinuria, without hematuria (Table 1, February 16). Due to suspected cutaneous-visceral loxoscelism, the patient was referred to our center for further evaluation. 

**Table 1:** Serial laboratory results from the initial presentation through hospitalization and first day in the pediatric intermediate care unit WBC: white blood cell; ANC: absolute neutrophil count; CRP: C-reactive protein; PT: prothrombin time; aPTT: activated partial thromboplastin time; INR: international normalized ratio; BUN: blood urea nitrogen; AST: aspartate aminotransferase; ALT: alanine aminotransferase; SGPT: gamma-glutamyl transpeptidase; IgA: immunoglobulin A: IgG: immunoglobulin G; IgE: immunoglobulin E; C3: C3 complement; C4: C4 complement

Test name	February 16	February 17 AM	February 17 PM	February 18	Normal range
Hemoglobin (g/dl)	12.3	12.1	11.7	10	11-13.5
Hematocrit (%)	34	34.7	33	28.2	33-39
WBC (×10^9^/L)	15.2	22.4	22.7	18.8	5.0-11.0
ANC (%)	90	97	95	95	25-70
Platelets (µL)	253,000	257,000	167,000	150,000	250,000-450,000
CRP (mg/dl)	2.4	6.4	30.5	22.1	0.3-1
PT (%)	-	59	44	53	78-110
aPTT	-	28	31	33	21-37
INR	-	1.3	1.7	1.45	0.97-1.2
Fibrinogen (mg/dl)	-	-	-	321	238-498
Creatinine (mg/dl)	0.4	0.69	0.54	0.69	0.25-1.0
BUN (mg/dl)	-	28	-	23	3-25
AST (U/L)	71	61	208	174	0-40
ALT (U/L)	34	40	174	199	4-35
GGT	24	28	96	133	9-48
IgA (mg/dl)	-	-	-	51	58-358
IgG (mg/dl)	-	-	-	338	596-1308
IgE (UI/ml)	-	-	-	11.5	0-87
C3 (mg/dl)	-	-	-	32	70-150
C4 (mg/dl)	-	-	-	5	13.5-45
Hemoglobinuria	(-)	(+)	-	(-)	Negative
Proteinuria (mg/dl)	15	Signs	30	30	Negative

He arrived at our emergency department at 4:00 AM on February 17, about 20 hours after symptom onset. On arrival, he remained in stable general condition, but continued to have persistent fever and fatigue. Repeat laboratory tests at that time showed increased leukocytosis up to 22,400/uL with a neutrophil count of 97%, CRP 6.4mg/dL, positive hemoglobinuria, and signs of proteinuria (all laboratory findings are summarized in Table 1, February 17 AM). Based on these findings, he was hospitalized with the presumptive diagnosis of loxoscelism.

**Figure 2:**
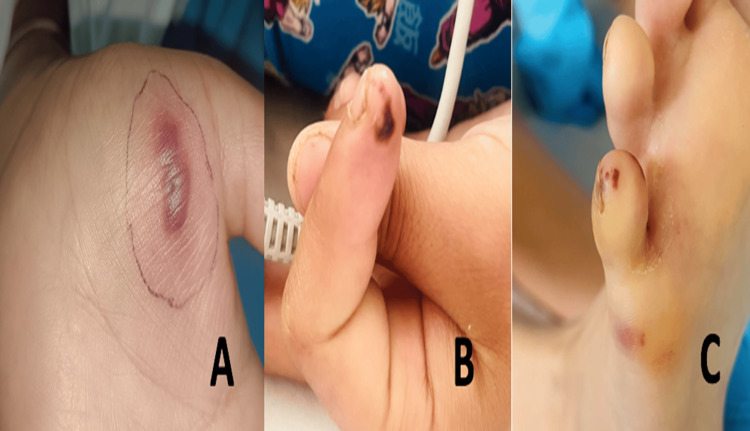
Evolution of cutaneous lesions after hospitalization: (A) enlargement of original hand lesion, (B) new purpuric lesions appearing on the fingertips, and (C) new purpuric lesions on the toes of the foot

Later that day, on February 17, during his stay in the general pediatric ward, the patient developed new purpuric lesions on the fingertips and heels, as well as progressive enlargement of the original hand lesion (Figure 2), along with worsening abdominal pain. Laboratory tests repeated at 9:00 PM on February 17 revealed elevated inflammatory markers, abnormal liver function tests, and coagulation abnormalities, with normal fibrinogen levels (Table 1, February 17 PM). Given clinical deterioration and laboratory progression, the patient was transferred the same night to the pediatric intermediate care unit (PICU), where empirical antibiotic therapy was initiated.

On February 18, in the PICU, the diagnostic evaluation was expanded. Connective tissue disease panels, including rheumatoid factor, antinuclear antibodies, antineutrophil cytoplasmic antibodies, and antiphospholipid antibodies, were all negative; however, hypogammaglobulinemia and low complement (C3, C4) were documented (Table 1, February 18). Microbiological studies revealed blood cultures positive for MRSA, and a skin biopsy of a purpuric lesion confirmed SV, showing superficial and deep perivascular neutrophilic infiltrates, necrosis of vessel walls with luminal thrombosis, and epidermal and dermal necrosis. No microorganisms were detected on Gram stain. Unfortunately, immunofluorescence was not performed.

Imaging studies revealed small splenic and left renal abscesses, as well as early osteomyelitis in the distal left femur and right tibia; other metastatic foci of infection, including endocarditis, were ruled out. Given the severity of the infection and these multiple foci, an underlying immunodeficiency was considered. The patient had no prior history of recurrent or severe infections. Initial hypogammaglobulinemia, low complement, and cytopenias seen on complete blood count normalized with infection resolution. Human immunodeficiency virus (HIV) and other secondary causes were ruled out, and follow-up immunologic testing (IgG subclasses, lymphocyte subpopulations) was normal.

The patient received six weeks of intravenous vancomycin monotherapy, starting at 40 mg/kg/day in four doses, adjusted to maintain therapeutic plasma levels. He improved rapidly, with fever resolution within 72 hours, negative follow-up blood cultures, normalization of laboratory parameters, and complete healing of skin lesions. He was discharged without functional sequelae and remained under follow-up with pediatric infectious diseases and immunology.

## Discussion

In an adult case series, cutaneous vasculitis associated with severe bacterial infections caused by diverse bacterial etiologic agents accounted for approximately 1.5% of all biopsy-confirmed vasculitis cases [1]. In a cohort of 32 patients with bacterial sepsis, skin lesions, and biopsy-proven septic vasculopathy, cutaneous manifestations were the initial event in 90.6% of cases, underscoring their potential diagnostic importance [10].

Although *Neisseria meningitidis* is the most frequently reported causative agent of SV, other bacteria, including *Neisseria gonorrhoeae,* *Staphylococcus aureus*, *Streptococcus pyogenes*, *Streptococcus pneumoniae*, *Pseudomonas *spp*.*, and *Rickettsia *spp., have also been implicated [2]. The pathophysiology of this entity involves multiple mechanisms such as disseminated intravascular coagulation, direct invasion of the microorganism into the vascular wall, immune-mediated mechanisms, septic embolism, and bacterial toxins, which may coexist in the same patient [11,12].

Clinically, SV often presents with palpable purpura, vesicles, and bullae in acral areas. Histologically, neutrophilic vasculitis and occlusive thrombi, composed of neutrophils, erythrocytes, platelets, and fibrin, can be observed, with or without detectable bacteria within vessels [10]. In our case, acral purpuric lesions were an early sign, initially misinterpreted as cutaneous-visceral loxoscelism. The progression of systemic symptoms and worsening cutaneous findings prompted further investigation. Timely skin biopsy and blood cultures confirmed the diagnosis of SV. Laboratory studies revealed hypogammaglobulinemia and hypocomplementemia; these findings may be explained within the pathophysiological framework of sepsis, in which systemic inflammation, driven by the activation of multiple inflammatory mediators, leads to endothelial activation, complement consumption, and adaptive immune responses involving B and T lymphocytes. This cascade contributes to immune dysregulation and, in severe cases, multiorgan dysfunction [12,13].

A few individual cases of SV associated with *S. aureus* have been reported. In adults, cutaneous vasculitis in the context of *S. aureus *sepsis has been documented in patients admitted to intensive care, based on clinical and microbiological evidence, though without histological confirmation [6,7]. In pediatric patients, one case reported histologically confirmed SV in a previously healthy child with MRSA sepsis, where consulting dermatologists played a crucial role in identifying disseminated infections by recognizing the skin manifestations and performing appropriate biopsies [8], while another case report described severe vascular injury in a child with CA-MRSA manifesting as an aortic aneurysm [14].

Larger studies have also demonstrated the relevance of cutaneous findings in bacteremia. In a retrospective analysis of 39 pediatric patients with *S. aureus* sepsis, no distinction was made between MRSA and methicillin-susceptible *Staphylococcus aureus* (MSSA), and no biopsies were performed. Skin manifestations were identified in six patients (15%), including maculopapular, petechial, purpuric, erythematous, pustular, and furuncular lesions. All six had concomitant systemic involvement, most commonly bronchopneumonia, and the presence of skin lesions appeared to correlate with higher mortality [15].

Similarly, a prospective cross-sectional study of 401 episodes of bacteremia in 375 hospitalized adults identified cutaneous manifestations in 34 patients (9%), with 27 (69%) classified as primary cutaneous lesions, most frequently cellulitis, lymphangitis, and superficial septic thrombophlebitis. Secondary manifestations were less common with seven cases (18%), including purpura fulminans (5%), vascular purpura (5%), livedo reticularis (2.5%), and septic emboli with erythematous nodules (2.5%) [16].

The presented case report adds to the limited pediatric literature by documenting biopsy-proven cutaneous SV secondary to CA-MRSA infection. It highlights an unusual but severe manifestation of invasive MRSA infection in children. Unlike more common causes of pediatric vasculitis that present with cutaneous purpura, such as Henoch-Schönlein purpura, lupus-associated purpura, or isolated leukocytoclastic vasculitis, our patient's immunologic and complement profiles, combined with microbiological and histopathological evidence, confirmed cutaneous vasculitis secondary to infection. This case underscores the importance of considering SV in the differential diagnosis of purpuric lesions in febrile children with systemic symptoms.

## Conclusions

SV in pediatrics is an infrequent yet critical manifestation of bacterial sepsis. It should be considered in the differential diagnosis of purpuric skin lesions in patients presenting with fever, purpuric lesions, and systemic symptoms, even in previously healthy children.

Cutaneous lesions may represent the first clinical sign of occult bacteremia and warrant immediate investigation, including blood cultures and skin biopsy. A multidisciplinary approach and a high clinical suspicion are essential for the timely diagnosis and management of this potentially life-threatening condition improving outcomes.
